# Supplementary material for: Predicting the burden of acute malnutrition in drought-prone regions of Kenya: A statistical analysis
Source: PLOS Glob Public Health. 2026 Apr 10;6(4):e0004485. doi: 10.1371/journal.pgph.0004485 (PMC13068232; doi:10.1371/journal.pgph.0004485)
Supplement: S1 Appendix — (DOCX) [file pgph.0004485.s001.docx]

Predicting the burden of acute malnutrition in drought-prone regions of Kenya: a statistical analysis

**S1 APPENDIX**

Description of anthropometric survey data

Table A. Effective sample size of anthropometric survey datasets available for analysis, by county, survey stratum and date. Pink-shaded survey are those that fall within the drought period (October 2016 to December 2017).

| **County** | **Date** | **Stratum** | **Subcounties within stratum** | **Number of observations** | | | | |
| --- | --- | --- | --- | --- | --- | --- | --- | --- |
|  |  |  |  | Total | Age 6-59mths | Complete observations† | Plausible WHZ‡ | Plausible WHZ, subcounty known |
| Baringo | Aug-15 | 1 | Tiaty | 670 | 669 | 667 | 666 | 666 |
|  | Jul-16 | 1 | Tiaty | 664 | 664 | 664 | 661 | 661 |
|  | Jul-17 | 1 | Tiaty | 334 | 334 | 332 | 329 | 329 |
|  | Jul-18 | 1 | Tiaty | 653 | 653 | 653 | 652 | 652 |
|  | Jul-18 | 2 | Baringo North, Baringo South | 580 | 574 | 580 | 571 | 571 |
| Garissa | Jun-16 | 1 | whole county | 809 | 809 | 809 | 809 | 809 |
|  | Jul-17 | 1 | whole county | 791 | 791 | 791 | 789 | 789 |
|  | Jul-18 | 1 | whole county | 926 | 924 | 926 | 924 | 924 |
| Isiolo | Jan-15 | 1 | whole county | 576 | 576 | 576 | 576 | 576 |
|  | Feb-16 | 1 | whole county | 496 | 496 | 496 | 496 | 496 |
|  | Jan-17 | 1 | whole county | 520 | 501 | 520 | 499 | 499 |
|  | Feb-18 | 1 | whole county | 610 | 610 | 610 | 605 | 605 |
|  | Jan-19 | 1 | whole county | 569 | 568 | 569 | 566 | 566 |
| Mandera | Jul-16 | 1 | whole county | 707 | 707 | 705 | 701 | 701 |
|  | Jul-17 | 1 | whole county | 779 | 779 | 779 | 776 | 776 |
|  | Jul-18 | 1 | whole county | 688 | 685 | 688 | 685 | 685 |
|  | Jul-19 | 1 | whole county | 666 | 666 | 666 | 664 | 664 |
| Marsabit | Aug-16 | 1 | Laisamis | 454 | 453 | 454 | 449 | 449 |
|  | Aug-16 | 2 | Moyale | 386 | 386 | 386 | 384 | 384 |
|  | Aug-16 | 3 | North Horr | 393 | 391 | 393 | 391 | 391 |
|  | Aug-16 | 4 | Saku | 298 | 298 | 298 | 296 | 296 |
|  | Jul-17 | 1 | Laisamis | 408 | 408 | 408 | 408 | 408 |
|  | Jul-17 | 2 | Moyale | 358 | 358 | 358 | 357 | 357 |
|  | Jul-17 | 3 | North Horr | 408 | 408 | 408 | 404 | 404 |
|  | Jul-17 | 4 | Saku | 248 | 248 | 248 | 247 | 247 |
|  | Jan-18 | 1 | Laisamis | 527 | 527 | 527 | 523 | 523 |
|  | Jan-18 | 3 | North Horr | 445 | 445 | 445 | 442 | 442 |
|  | Jul-18 | 1 | Laisamis | 391 | 391 | 391 | 386 | 386 |
|  | Jul-18 | 2 | Moyale | 406 | 406 | 406 | 404 | 404 |
|  | Jul-18 | 3 | North Horr | 346 | 346 | 346 | 343 | 343 |
|  | Jul-18 | 4 | Saku | 366 | 366 | 366 | 364 | 364 |
|  | Jul-19 | 1 | Laisamis | 503 | 503 | 503 | 501 | 501 |
|  | Jul-19 | 2 | Moyale | 403 | 403 | 403 | 402 | 402 |
|  | Jul-19 | 3 | North Horr | 398 | 396 | 398 | 394 | 394 |
|  | Jul-19 | 4 | Saku | 216 | 214 | 216 | 214 | 214 |
| Samburu | Jun-16 | 1 | whole county | 550 | 550 | 547 | 546 | 546 |
|  | Jun-17 | 1 | whole county | 532 | 530 | 530 | 525 | 525 |
|  | Jun-18 | 1 | whole county | 510 | 510 | 510 | 510 | 510 |
|  | Jun-19 | 1 | whole county | 504 | 504 | 504 | 502 | 502 |
| Tana River | Jul-16 | 1 | whole county | 546 | 546 | 541 | 538 | 538 |
|  | Feb-18 | 1 | whole county | 639 | 639 | 639 | 636 | 636 |
|  | Feb-19 | 1 | whole county | 662 | 660 | 662 | 655 | 655 |
| Turkana | Jun-16 | 1 | Turkana North | 667 | 667 | 665 | 662 | 662 |
|  | Jun-16 | 2 | Loima, Turkana Central | 672 | 672 | 672 | 669 | 669 |
|  | Jun-16 | 3 | Turkana East, Turkana South | 841 | 841 | 837 | 835 | 835 |
|  | Jun-16 | 4 | Turkana West | 572 | 572 | 569 | 567 | 567 |
|  | Jan-17 | 1 | Kibish, Turkana North | 726 | 726 | 726 | 718 | 718 |
|  | Jan-17 | 2 | Loima, Turkana Central | 814 | 814 | 814 | 804 | 804 |
|  | Jan-17 | 3 | Turkana East, Turkana South | 397 | 397 | 397 | 393 | 393 |
|  | Jan-17 | 4 | Turkana West | 492 | 492 | 492 | 491 | 491 |
|  | Jun-17 | 1 | Kibish, Turkana North | 637 | 636 | 637 | 632 | 632 |
|  | Jun-17 | 2 | Loima, Turkana Central | 658 | 658 | 658 | 653 | 653 |
|  | Jun-17 | 3 | Turkana East, Turkana South | 571 | 571 | 571 | 567 | 567 |
|  | Jun-17 | 4 | Turkana West | 525 | 525 | 525 | 522 | 522 |
|  | Feb-18 | 1 | Kibish, Turkana North | 490 | 489 | 490 | 487 | 487 |
|  | Feb-18 | 2 | Loima, Turkana Central | 611 | 610 | 611 | 603 | 603 |
|  | Feb-18 | 3 | Turkana East, Turkana South | 725 | 723 | 725 | 721 | 721 |
|  | Feb-18 | 4 | Turkana West | 690 | 688 | 690 | 687 | 687 |
|  | Jun-18 | 1 | Kibish, Turkana North | 576 | 576 | 576 | 573 | 573 |
|  | Jun-18 | 2 | Loima, Turkana Central | 558 | 558 | 558 | 555 | 555 |
|  | Jun-18 | 3 | Turkana East, Turkana South | 600 | 600 | 600 | 595 | 595 |
|  | Jun-18 | 4 | Turkana West | 688 | 688 | 688 | 685 | 685 |
|  | Jun-19 | 1 | Kibish, Turkana North | 544 | 541 | 544 | 539 | 539 |
|  | Jun-19 | 2 | Loima, Turkana Central | 367 | 367 | 367 | 367 | 367 |
|  | Jun-19 | 3 | Turkana East, Turkana South | 502 | 502 | 502 | 502 | 502 |
|  | Jun-19 | 4 | Turkana West | 693 | 693 | 693 | 693 | 693 |
| Wajir | Jun-15 | 1 | Eldas, Habaswein, Tarbaj, Wajir East, Wajir South, Wajir West | 518 | 518 | 518 | 513 | 513 |
|  | Jun-15 | 2 | Wajir North | 498 | 498 | 498 | 493 | 493 |
|  | Jul-16 | 1 | Eldas, Tarbaj, Wajir East, Wajir South, Wajir West | 574 | 574 | 574 | 573 | 573 |
|  | Jul-16 | 2 | Wajir North | 665 | 665 | 665 | 664 | 664 |
|  | Jul-17 | 1 | Eldas, Habaswein, Tarbaj, Wajir East, Wajir South, Wajir West | 597 | 595 | 597 | 594 | 594 |
|  | Jul-17 | 2 | Wajir North | 728 | 726 | 728 | 726 | 726 |
|  | Jul-18 | 1 | Eldas, Tarbaj, Wajir East, Wajir South, Wajir West | 737 | 732 | 737 | 731 | 731 |
|  | Jul-18 | 2 | Wajir North | 670 | 666 | 670 | 664 | 664 |
|  | Jun-19 | 1 | whole county | 825 | 816 | 819 | 814 | 814 |
| West Pokot | Jun-17 | 1 | whole county | 540 | 540 | 540 | 538 | 538 |
|  | Jun-18 | 1 | whole county | 647 | 615 | 619 | 615 | 615 |
|  | Jul-19 | 1 | whole county | 532 | 532 | 532 | 531 | 531 |
| **Totals** |  |  |  | **44,082** | **43,975** | **44,022** | **43,766** | **43,766** |

† Age in months, sex, weight in Kg, height in cm, oedema presence all non-missing. ‡ Weight-for-height Z-score within the range -5 to +5.

Table B. Point estimates, 95% confidence intervals and design effect of the prevalence of global acute malnutrition, by county, survey stratum and date. Pink-shaded survey are those that fall within the drought period (October 2016 to December 2017).

| **County** | **Date** | **Stratum** | **Subcounties within stratum** | **SAM prevalence (95%CI)** | **SAM design effect** | **GAM prevalence (95%CI)** | **GAM design effect** |
| --- | --- | --- | --- | --- | --- | --- | --- |
| Baringo | Aug-15 | 1 | Baringo East - Tiaty | 3.8% (2.4% to 5.8%) | 1.34 | 18.6% (15.3% to 22.5%) | 1.47 |
|  | Jul-16 | 1 | Baringo East - Tiaty | 3.8% (2.5% to 5.7%) | 1.21 | 22.4% (18.5% to 26.8%) | 1.70 |
|  | Jul-17 | 1 | Baringo East - Tiaty | 5.2% (3.1% to 8.5%) | 1.17 | 24.6% (19.4% to 30.7%) | 1.47 |
|  | Jul-18 | 1 | Baringo East - Tiaty | 4.3% (3.0% to 6.1%) | 0.92 | 16.9% (14.0% to 20.2%) | 1.15 |
|  | Jul-18 | 2 | Baringo North, Baringo South | 0.9% (0.4% to 2.0%) | 0.96 | 8.4% (5.9% to 11.9%) | 1.71 |
| Garissa | Jun-16 | 1 | whole county | 2.7% (1.8% to 4.0%) | 0.94 | 15.1% (12.3% to 18.3%) | 1.49 |
|  | Jul-17 | 1 | whole county | 1.5% (0.9% to 2.4%) | 0.71 | 16.0% (12.9% to 19.5%) | 1.65 |
|  | Jul-18 | 1 | whole county | 2.4% (1.6% to 3.5%) | 0.9 | 14.0% (11.5% to 16.9%) | 1.46 |
| Isiolo | Jan-15 | 1 | whole county | 1.7% (1.0% to 3.0%) | 0.76 | 13.0% (10.8% to 15.6%) | 0.77 |
|  | Feb-16 | 1 | whole county | 1.2% (0.4% to 3.2%) | 1.56 | 12.1% (9.4% to 15.5%) | 1.13 |
|  | Jan-17 | 1 | whole county | 3.8% (2.5% to 5.7%) | 0.84 | 20.0% (16.3% to 24.3%) | 1.29 |
|  | Feb-18 | 1 | whole county | 2.3% (1.4% to 3.7%) | 0.84 | 13.4% (10.8% to 16.5%) | 1.11 |
|  | Jan-19 | 1 | whole county | 1.1% (0.4% to 2.5%) | 1.22 | 9.4% (6.8% to 12.8%) | 1.51 |
| Mandera | Jul-16 | 1 | whole county | 4.7% (3.1% to 7.1%) | 1.53 | 22.4% (18.5% to 26.9%) | 1.85 |
|  | Jul-17 | 1 | whole county | 5.4% (3.8% to 7.7%) | 1.45 | 25.3% (21.5% to 29.4%) | 1.65 |
|  | Jul-18 | 1 | whole county | 3.2% (2.1% to 4.9%) | 1.09 | 16.8% (13.7% to 20.4%) | 1.40 |
|  | Jul-19 | 1 | whole county | 5.0% (3.2% to 7.7%) | 1.81 | 22.1% (18.3% to 26.5%) | 1.71 |
| Marsabit | Aug-16 | 1 | Laisamis | 5.3% (3.2% to 8.8%) | 1.66 | 23.2% (18.9% to 28.1%) | 1.40 |
|  | Aug-16 | 2 | Moyale | 5.9% (3.8% to 8.9%) | 1.19 | 24.8% (20.3% to 29.8%) | 1.26 |
|  | Aug-16 | 3 | North Horr | 3.6% (2.4% to 5.4%) | 0.79 | 20.7% (17.0% to 24.8%) | 1.25 |
|  | Aug-16 | 4 | Saku | 2.6% (1.2% to 5.3%) | 1.41 | 13.7% (9.9% to 18.8%) | 1.67 |
|  | Jul-17 | 1 | Laisamis | 6.4% (4.2% to 9.6%) | 1.53 | 30.3% (25.7% to 35.4%) | 1.46 |
|  | Jul-17 | 2 | Moyale | 1.3% (0.6% to 2.8%) | 0.78 | 7.8% (5.6% to 10.8%) | 0.94 |
|  | Jul-17 | 3 | North Horr | 1.4% (0.5% to 3.6%) | 1.19 | 6.4% (4.0% to 10.2%) | 1.40 |
|  | Jul-17 | 4 | Saku | 1.2% (0.6% to 2.7%) | 0.83 | 7.7% (4.8% to 12.1%) | 1.86 |
|  | Jan-18 | 1 | Laisamis | 1.5% (0.6% to 3.5%) | 1.13 | 9.2% (6.4% to 13.1%) | 1.38 |
|  | Jan-18 | 3 | North Horr | 3.8% (2.0% to 7.3%) | 1.75 | 22.5% (17.5% to 28.4%) | 1.72 |
|  | Jul-18 | 1 | Laisamis | 5.4% (3.6% to 8.2%) | 1.02 | 30.9% (25.5% to 36.9%) | 1.60 |
|  | Jul-18 | 2 | Moyale | 5.2% (3.5% to 7.8%) | 1.03 | 21.5% (17.8% to 25.7%) | 1.06 |
|  | Jul-18 | 3 | North Horr | 3.5% (2.3% to 5.4%) | 0.61 | 23.0% (19.0% to 27.6%) | 0.92 |
|  | Jul-18 | 4 | Saku | 3.0% (1.7% to 5.4%) | 1.07 | 24.9% (20.0% to 30.5%) | 1.54 |
|  | Jul-19 | 1 | Laisamis | 1.7% (0.7% to 3.9%) | 0.95 | 7.4% (4.9% to 11.1%) | 1.05 |
|  | Jul-19 | 2 | Moyale | 0.4% (0.1% to 2.8%) | 1.01 | 8.1% (5.1% to 12.7%) | 1.20 |
|  | Jul-19 | 3 | North Horr | 0.8% (0.3% to 2.4%) | 0.92 | 6.0% (3.9% to 9.2%) | 1.12 |
|  | Jul-19 | 4 | Saku | 1.9% (0.8% to 4.4%) | 0.8 | 10.3% (5.3% to 19.1%) | 2.67 |
| Samburu | Jun-16 | 1 | whole county | 2.6% (1.5% to 4.2%) | 0.95 | 13.9% (11.5% to 16.8%) | 0.84 |
|  | Jun-17 | 1 | whole county | 3.6% (2.3% to 5.6%) | 1 | 18.1% (14.5% to 22.4%) | 1.45 |
|  | Jun-18 | 1 | whole county | 4.3% (2.8% to 6.6%) | 1.12 | 15.9% (12.6% to 19.8%) | 1.28 |
|  | Jun-19 | 1 | whole county | 2.4% (1.4% to 4.1%) | 0.98 | 15.7% (12.7% to 19.3%) | 1.08 |
| Tana River | Jul-16 | 1 | whole county | 2.2% (1.2% to 4.2%) | 1.29 | 14.5% (11.2% to 18.6%) | 1.57 |
|  | Feb-18 | 1 | whole county | 2.8% (1.6% to 4.9%) | 1.45 | 16.0% (12.0% to 21.1%) | 2.51 |
|  | Feb-19 | 1 | whole county | 3.4% (2.4% to 4.8%) | 0.74 | 15.1% (12.1% to 18.7%) | 1.45 |
| Turkana | Jun-16 | 1 | Turkana North | 4.2% (2.6% to 6.8%) | 1.77 | 23.3% (19.3% to 27.8%) | 1.75 |
|  | Jun-16 | 2 | Loima, Turkana Central | 8.1% (6.1% to 10.6%) | 1.2 | 30.4% (26.5% to 34.5%) | 1.43 |
|  | Jun-16 | 3 | Turkana East, Turkana South | 8.5% (6.4% to 11.3%) | 1.21 | 33.4% (29.6% to 37.4%) | 1.11 |
|  | Jun-16 | 4 | Turkana West | 2.5% (1.3% to 4.6%) | 1.29 | 16.0% (12.1% to 20.9%) | 1.83 |
|  | Jan-17 | 1 | Kibish, Turkana North | 3.3% (2.1% to 5.3%) | 1.1 | 15.7% (12.1% to 20.2%) | 1.82 |
|  | Jan-17 | 2 | Loima, Turkana Central | 7.6% (4.8% to 11.7%) | 2.27 | 30.1% (24.5% to 36.2%) | 2.29 |
|  | Jan-17 | 3 | Turkana East, Turkana South | 6.1% (4.5% to 8.3%) | 1.09 | 24.1% (19.5% to 29.3%) | 2.32 |
|  | Jan-17 | 4 | Turkana West | 6.7% (4.6% to 9.7%) | 2.09 | 26.0% (21.9% to 30.5%) | 2.03 |
|  | Jun-17 | 1 | Kibish, Turkana North | 8.3% (6.2% to 11.0%) | 1.3 | 30.6% (27.0% to 34.5%) | 1.10 |
|  | Jun-17 | 2 | Loima, Turkana Central | 3.0% (1.9% to 4.7%) | 0.99 | 17.2% (13.7% to 21.5%) | 1.65 |
|  | Jun-17 | 3 | Turkana East, Turkana South | 4.7% (3.2% to 6.9%) | 1.06 | 17.3% (14.1% to 21.0%) | 1.21 |
|  | Jun-17 | 4 | Turkana West | 3.5% (1.9% to 6.5%) | 1.33 | 20.7% (15.8% to 26.6%) | 1.69 |
|  | Feb-18 | 1 | Kibish, Turkana North | 9.3% (7.5% to 11.6%) | 1.05 | 30.2% (26.5% to 34.1%) | 1.47 |
|  | Feb-18 | 2 | Loima, Turkana Central | 5.9% (4.0% to 8.5%) | 0.92 | 22.9% (19.2% to 27.1%) | 0.91 |
|  | Feb-18 | 3 | Turkana East, Turkana South | 12.3% (10.0% to 15.1%) | 0.87 | 36.7% (32.2% to 41.4%) | 1.34 |
|  | Feb-18 | 4 | Turkana West | 2.4% (1.3% to 4.2%) | 1.49 | 16.1% (13.4% to 19.2%) | 1.15 |
|  | Jun-18 | 1 | Kibish, Turkana North | 2.7% (1.6% to 4.4%) | 1.03 | 19.5% (15.9% to 23.7%) | 1.48 |
|  | Jun-18 | 2 | Loima, Turkana Central | 7.8% (5.3% to 11.2%) | 1.54 | 30.9% (25.2% to 37.2%) | 2.20 |
|  | Jun-18 | 3 | Turkana East, Turkana South | 2.3% (1.4% to 3.7%) | 0.81 | 14.8% (11.6% to 18.7%) | 1.47 |
|  | Jun-18 | 4 | Turkana West | 4.3% (2.5% to 7.3%) | 1.69 | 15.9% (12.1% to 20.6%) | 1.74 |
|  | Jun-19 | 1 | Kibish, Turkana North | 7.5% (5.3% to 10.5%) | 1.29 | 24.1% (19.5% to 29.5%) | 1.86 |
|  | Jun-19 | 2 | Loima, Turkana Central | 2.6% (1.7% to 4.0%) | 0.91 | 15.6% (12.7% to 19.0%) | 1.32 |
|  | Jun-19 | 3 | Turkana East, Turkana South | 5.5% (3.8% to 8.0%) | 1.42 | 19.0% (15.2% to 23.4%) | 1.92 |
|  | Jun-19 | 4 | Turkana West | 6.5% (4.3% to 9.6%) | 2.02 | 23.1% (19.6% to 27.0%) | 1.38 |
| Wajir | Jun-15 | 1 | Eldas, Habaswein, Tarbaj, Wajir East, Wajir South, Wajir West | 2.5% (1.5% to 4.2%) | 0.89 | 17.9% (14.9% to 21.5%) | 1.00 |
|  | Jun-15 | 2 | Wajir North | 2.8% (1.9% to 4.1%) | 0.67 | 14.0% (10.6% to 18.1%) | 1.72 |
|  | Jul-16 | 1 | Eldas, Tarbaj, Wajir East, Wajir South, Wajir West | 2.5% (1.4% to 4.4%) | 1.25 | 16.0% (12.5% to 20.3%) | 1.77 |
|  | Jul-16 | 2 | Wajir North | 2.3% (1.5% to 3.6%) | 0.86 | 13.1% (10.7% to 16.0%) | 1.13 |
|  | Jul-17 | 1 | Eldas, Habaswein, Tarbaj, Wajir East, Wajir South, Wajir West | 2.6% (1.7% to 3.9%) | 1.02 | 16.2% (13.5% to 19.4%) | 1.33 |
|  | Jul-17 | 2 | Wajir North | 2.2% (1.4% to 3.6%) | 0.64 | 14.2% (11.4% to 17.5%) | 0.95 |
|  | Jul-18 | 1 | Eldas, Tarbaj, Wajir East, Wajir South, Wajir West | 1.7% (0.9% to 3.0%) | 1.09 | 9.3% (7.4% to 11.7%) | 0.96 |
|  | Jul-18 | 2 | Wajir North | 2.9% (1.6% to 5.0%) | 1.77 | 16.8% (13.4% to 20.9%) | 1.90 |
|  | Jun-19 | 1 | whole county | 2.1% (1.1% to 3.9%) | 1.39 | 10.8% (8.2% to 14.2%) | 1.58 |
| West Pokot | Jun-17 | 1 | whole county | 3.7% (2.4% to 5.8%) | 1.09 | 20.4% (16.8% to 24.7%) | 1.33 |
|  | Jun-18 | 1 | whole county | 1.5% (0.8% to 2.8%) | 1.01 | 11.4% (9.2% to 14.0%) | 0.92 |
|  | Jul-19 | 1 | whole county | 2.4% (1.4% to 4.3%) | 1.12 | 12.1% (8.9% to 16.1%) | 1.66 |

Predictor selection and patterns


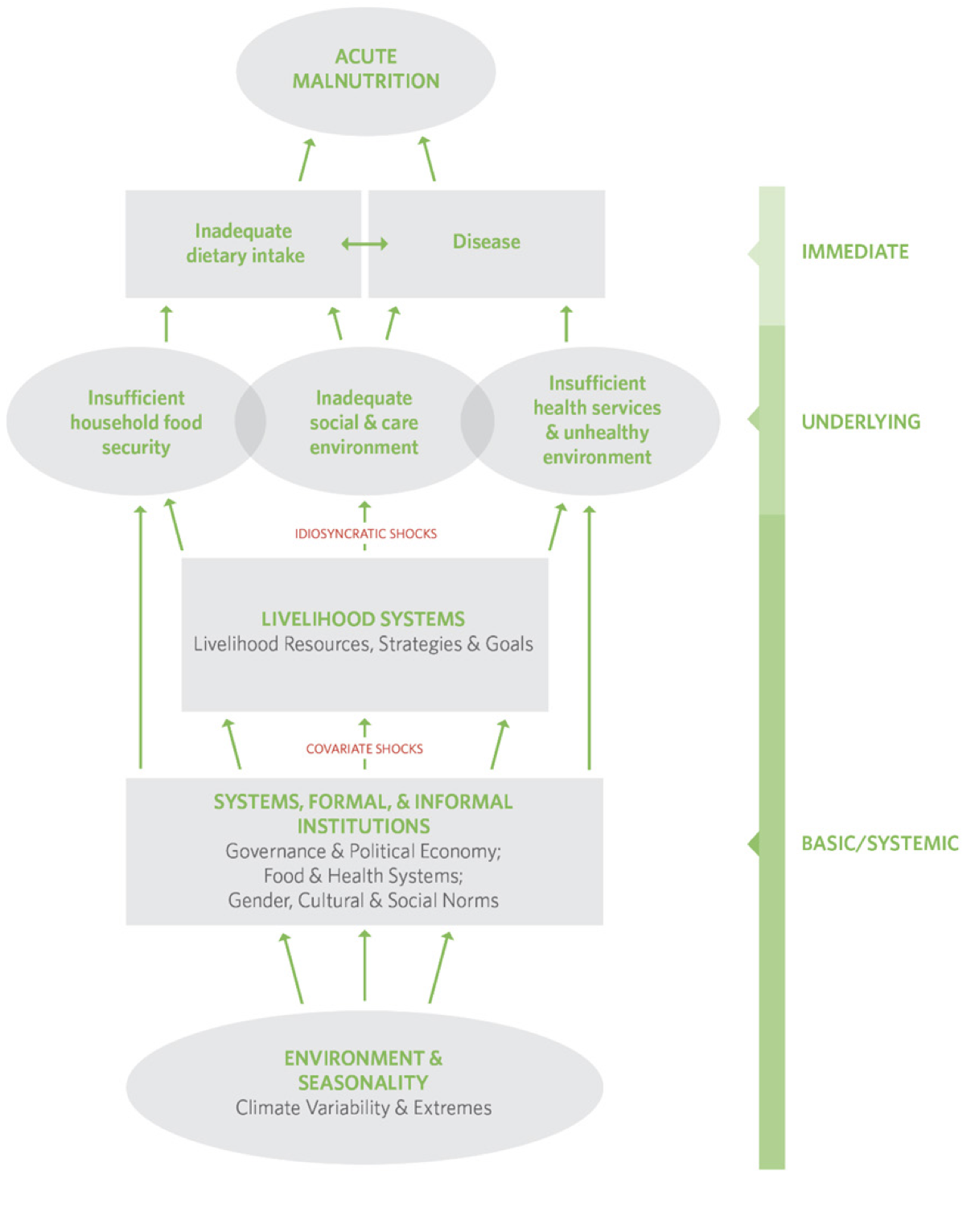


Fig A. Proposed causal framework of acute malnutrition determinants in African dry lands (22).

Fig B. Causal framework of acute malnutrition in children used in a previous statistical modelling study (18).

Table C. Summary of predictor variable average values and ranges.

| **Variable** | **Scale** | **Percent of non-zero values** | **Mean** | **Median** | **Minimum** | **Maximum** |
| --- | --- | --- | --- | --- | --- | --- |
| Drought conditions |  |  |  |  |  |  |
| Standardised precipitation index (3mths mean) | Sub-county | 100.0% | 0.2 | 0.1 | -1.4 | 2.7 |
| Standardised precipitation index (6mths mean) | Sub-county | 100.0% | 0.1 | 0.1 | -1.1 | 2.1 |
| Normalised difference vegetation index (3mths mean) | Sub-county | 100.0% | 0.3 | 0.2 | 0.1 | 0.7 |
| Normalised difference vegetation index (6mths mean) | Sub-county | 100.0% | 0.3 | 0.2 | 0.1 | 0.7 |
| Standardised normalised difference vegetation index (3mths mean) | Sub-county | 45.5% | 0.2 | 0.0 | 0.0 | 1.4 |
| Standardised normalised difference vegetation index (3mths mean) | Sub-county | 60.1% | 0.1 | 0.1 | 0.0 | 0.7 |
| Food security |  |  |  |  |  |  |
| Price per Kg of white maize at the nearest market (KSH) (3mths mean) | Sub-county | 100.0% | 71.2 | 68.5 | 32.5 | 128.1 |
| Price per Kg of white maize at the nearest market (KSH) (6mths mean) | Sub-county | 100.0% | 71.2 | 68.3 | 34.4 | 123 |
| Observed incidence of acute malnutrition† |  |  |  |  |  |  |
| Incidence of SAM treatment admissions (3mths mean) | Sub-county | 99.1% | 34.6 | 26.4 | 0.0 | 301.4 |
| Incidence of SAM treatment admissions (6mths mean) | Sub-county | 99.6% | 34.7 | 26.5 | 0.0 | 281.2 |
| Incidence of MAM treatment admissions (3mths mean) | Sub-county | 99.3% | 99.2 | 62.6 | 0.0 | 1063.9 |
| Incidence of MAM treatment admissions (6mths mean) | Sub-county | 99.7% | 99.3 | 62 | 0.0 | 716.4 |
| Occurrence of epidemic disease† |  |  |  |  |  |  |
| Suspected cholera incidence rate (3mths mean) | County | 25.6% | 0.6 | 0.0 | 0.0 | 35.4 |
| Suspected cholera incidence rate (6mths mean) | County | 29.4% | 0.7 | 0.0 | 0.0 | 30.8 |
| Suspected measles incidence rate (3mths mean) | County | 40.4% | 0.3 | 0.0 | 0.0 | 12.4 |
| Suspected measles incidence rate (6mths mean) | County | 54.4% | 0.3 | 0.0 | 0.0 | 6.7 |
| Utilisation of health services† |  |  |  |  |  |  |
| Number of third doses of pentavalent vaccine administered per population (3mths mean) | County | 100.0% | 172.2 | 185.9 | 37.6 | 354.5 |
| Number of third doses of pentavalent vaccine administered per population (6mths mean) | County | 100.0% | 171.4 | 187.5 | 41.6 | 338.5 |
| Number of first doses of measles, mumps and rubella vaccine administered per population (3mths mean) | County | 100.0% | 170.8 | 181.7 | 41.4 | 355.3 |
| Number of first doses of measles, mumps and rubella vaccine administered per population (9mths mean) | County | 100.0% | 170.1 | 185.8 | 44.4 | 316.9 |
| Proportion of the population with access to safe assisted births (static) | Sub-county | 100.0% | 0.5 | 0.6 | 0.3 | 0.7 |
| Wider social conditions |  |  |  |  |  |  |
| Proportion of the female population aged 15-49yo that is literate (static) | Sub-county | 100.0% | 0.3 | 0.3 | 0.1 | 0.8 |
| Proportion of children attending primary education (static) | County | 100.0% | 0.3 | 0.2 | 0.0 | 0.4 |
| Incidence of insecurity events (3mths mean) † | Sub-county | 30.8% | 0.1 | 0.0 | 0.0 | 3.0 |
| Incidence of insecurity events (6mths mean) † | Sub-county | 45.6% | 0.1 | 0.0 | 0.0 | 2.0 |
| Incidence of insecurity fatalities (3mths mean) † | Sub-county | 23.7% | 0.3 | 0.0 | 0.0 | 28.4 |
| Incidence of insecurity fatalities (3mths mean) † | Sub-county | 34.7% | 0.3 | 0.0 | 0.0 | 14.2 |

† All indicators are expressed per 100,000 population per month.


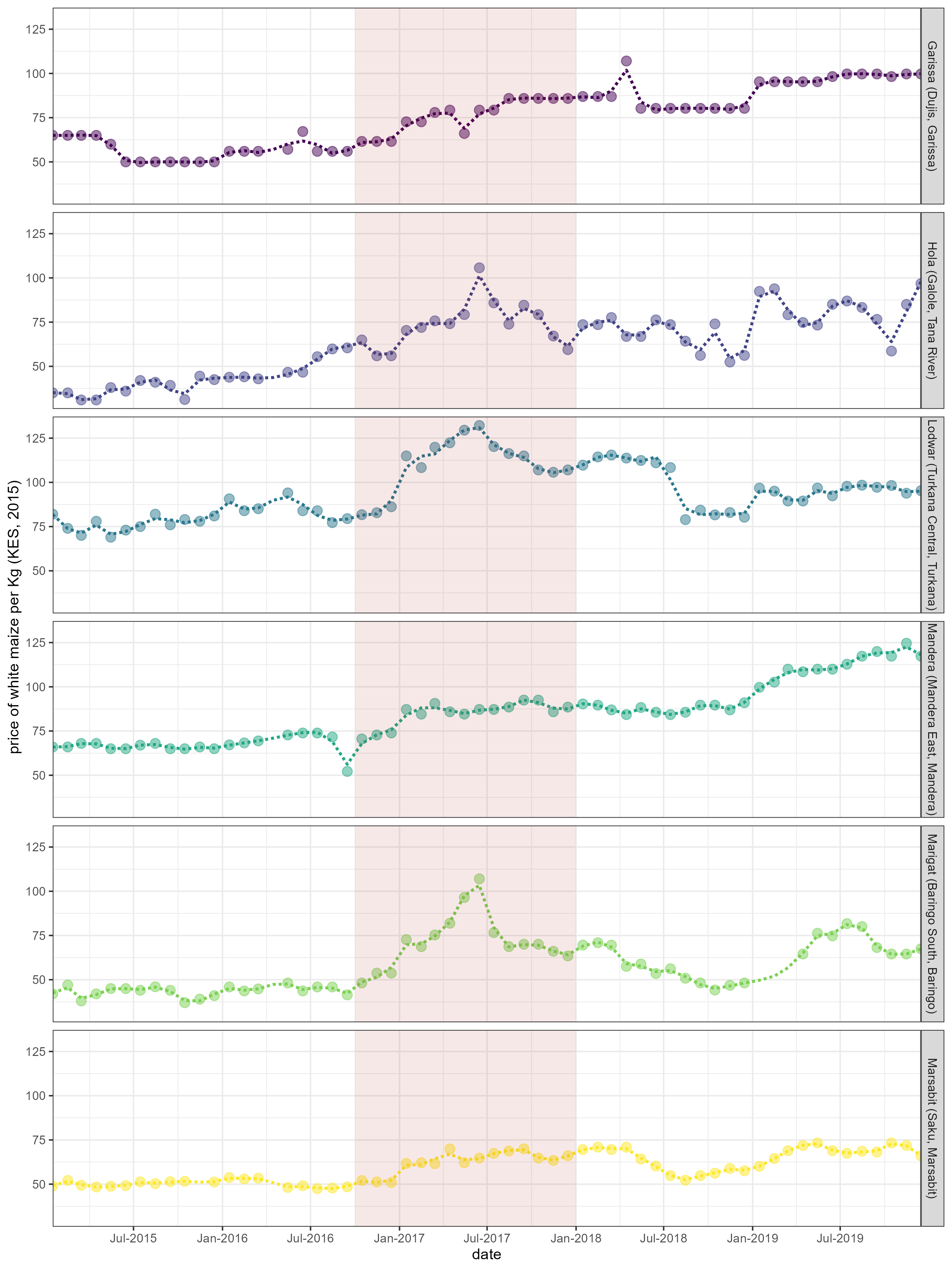


Fig C. Time series of the price of white maize in 2015 KSH equivalents, adjusted for inflation, within each of the five sentinel markets with consistent data availability. The pink-shaded band indicates the drought period (October 2016 to December 2017).

Univariate analysis


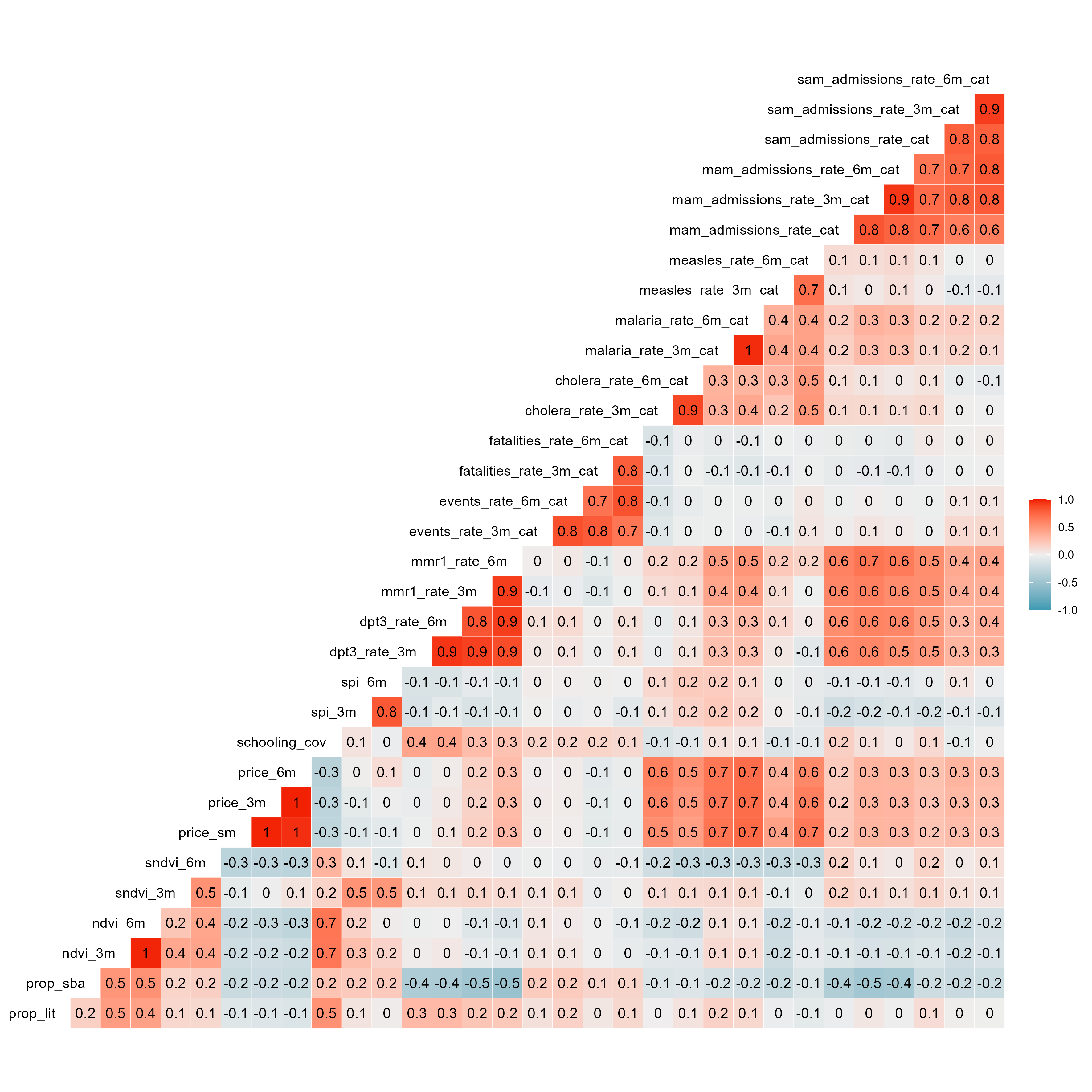


Fig D. Two-way Pearson correlation coefficients between pairs of predictor variables. The extent of shading indicates the strength of positive (red-coloured) or negative (teal-coloured) correlation.


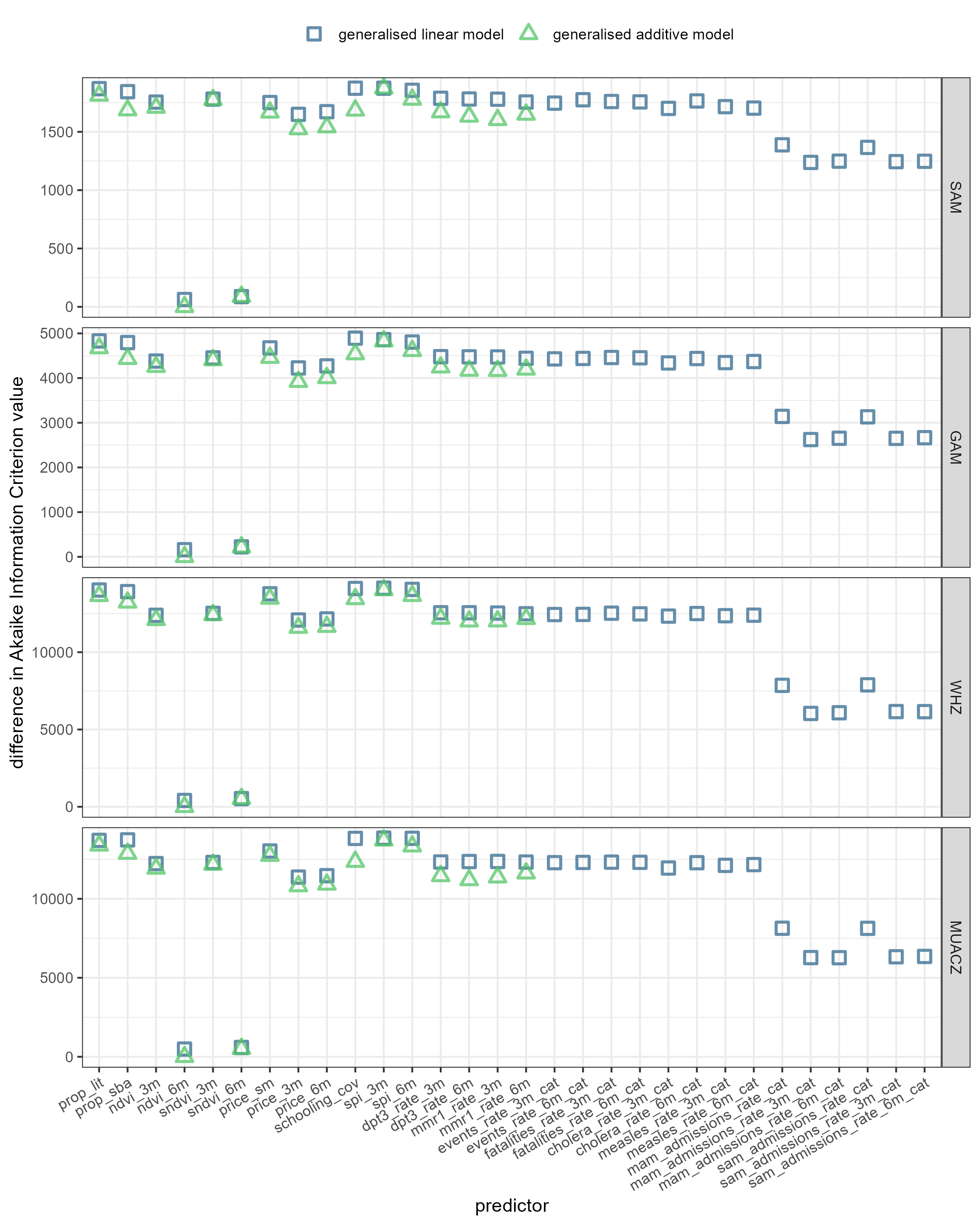


Fig E. Goodness of fit of univariate generalised linear and additive (for continuous variables only) models, by outcome and predictor variable. The y-axis reports the difference between the Akaike Information Criterion (AIC) of each variable and the AIC of the variable with lowest AIC, i.e. best-fitting. Lower AIC differences thus indicate better fit.

Predictive model performance


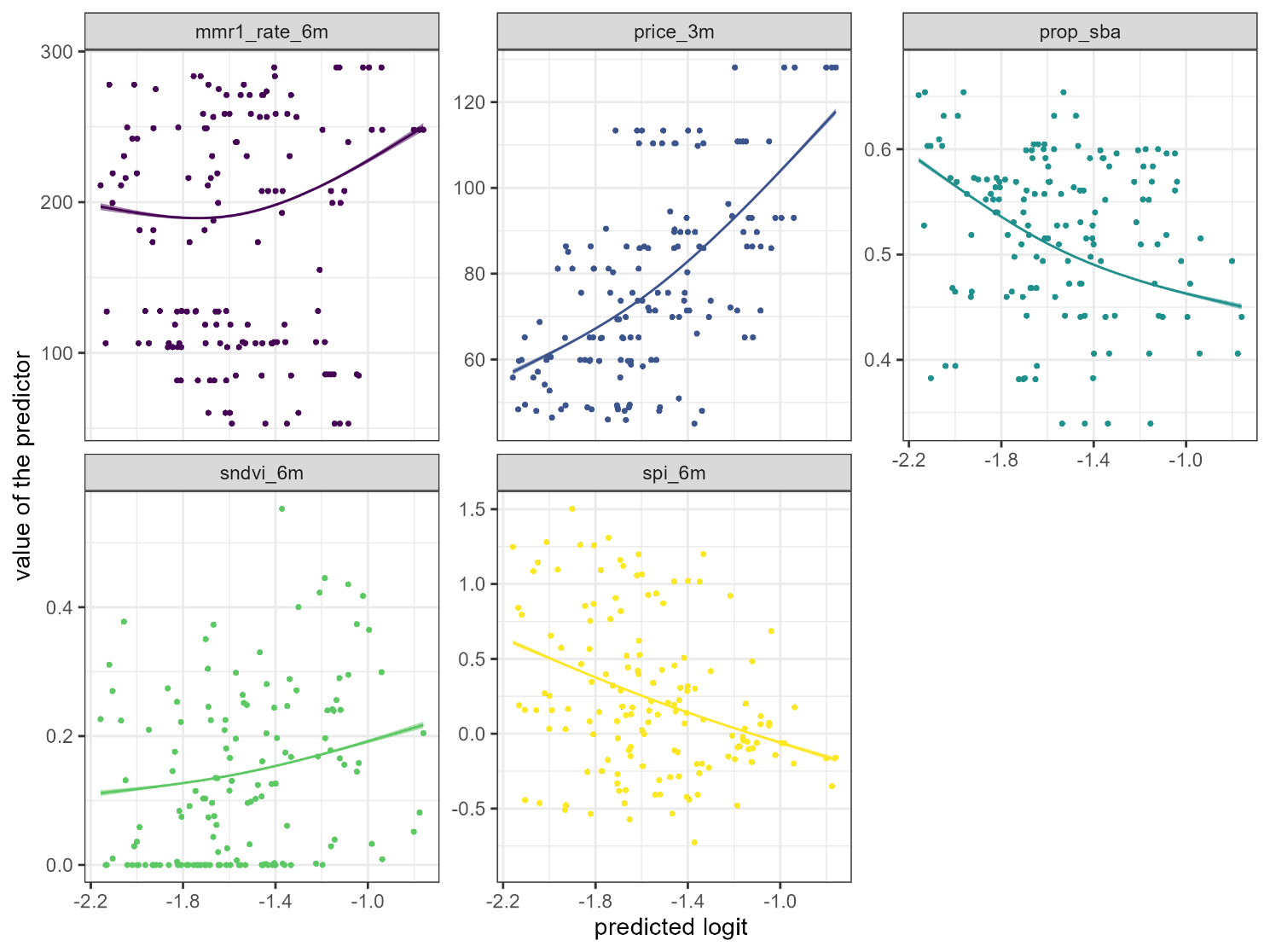


Fig F. Correlation between predicted logits and values of each continuous predictor, for a GLM model of GAM. Lines indicate smoothed trends based on additive models with 3 knots.


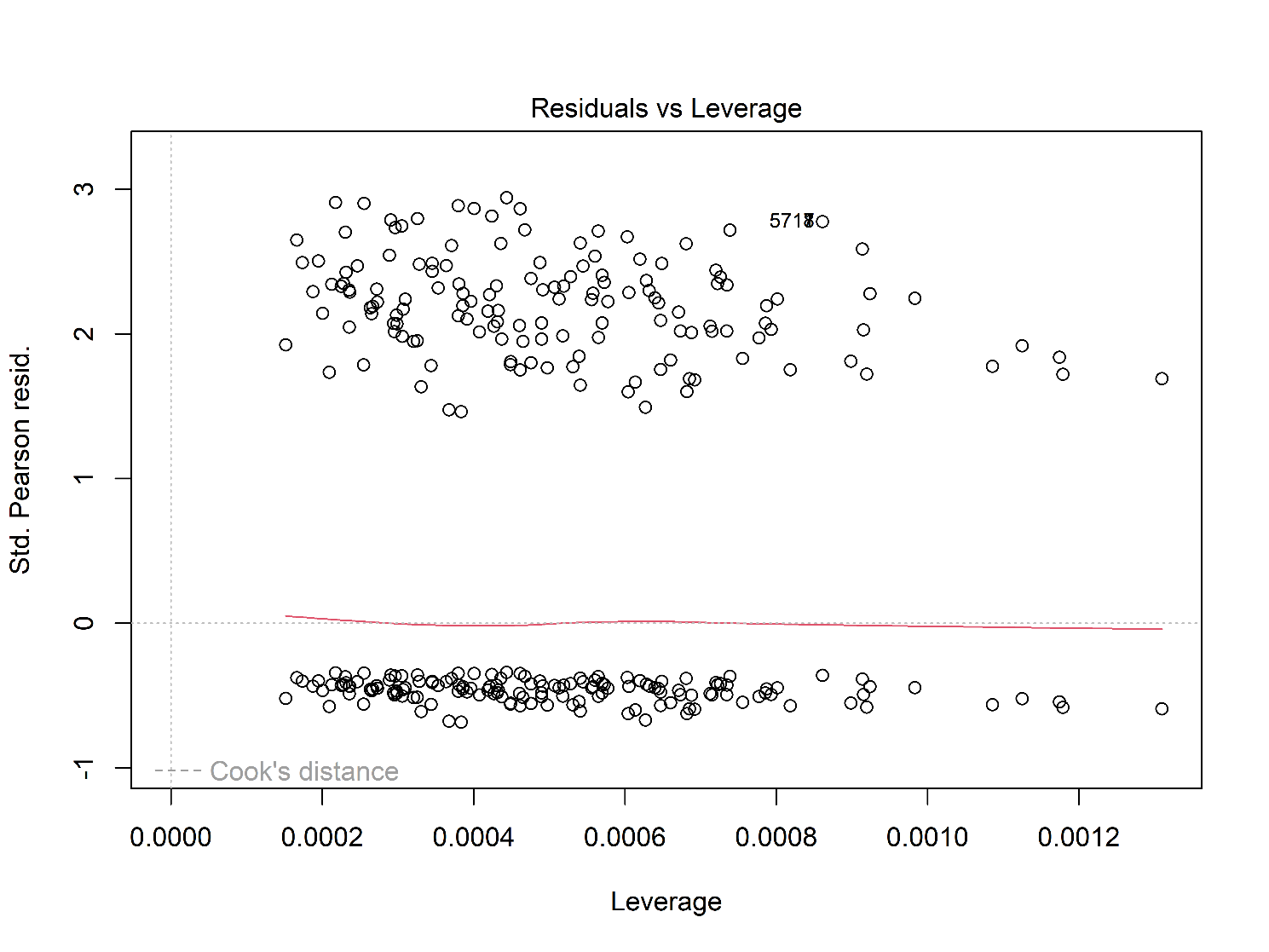


Fig G. Graph of observation leverage versus residuals, for a GLM model of GAM.

Table D. Multicollinearity tests for a GLM model of GAM. Standardised GVIF values ≥ 5 typically suggest collinearity.

| Predictor | Generalised variance inflation factor (GVIF) | Degrees of freedom (Df) | Standardised GVIF GVIF^(1/(2*Df)) |
| --- | --- | --- | --- |
| sndvi_6m | 1.41042 | 1 | 1.187611 |
| mam_admissions_rate_3m_cat | 3.620132 | 3 | 1.239141 |
| price_3m | 3.088774 | 1 | 1.757491 |
| cholera_rate_3m_cat | 5.899254 | 4 | 1.248388 |
| events_rate_3m_cat | 1.831223 | 4 | 1.078556 |
| mmr1_rate_6m | 2.996897 | 1 | 1.731155 |
| prop_sba | 1.976271 | 1 | 1.405799 |
| spi_6m | 1.476377 | 1 | 1.215063 |


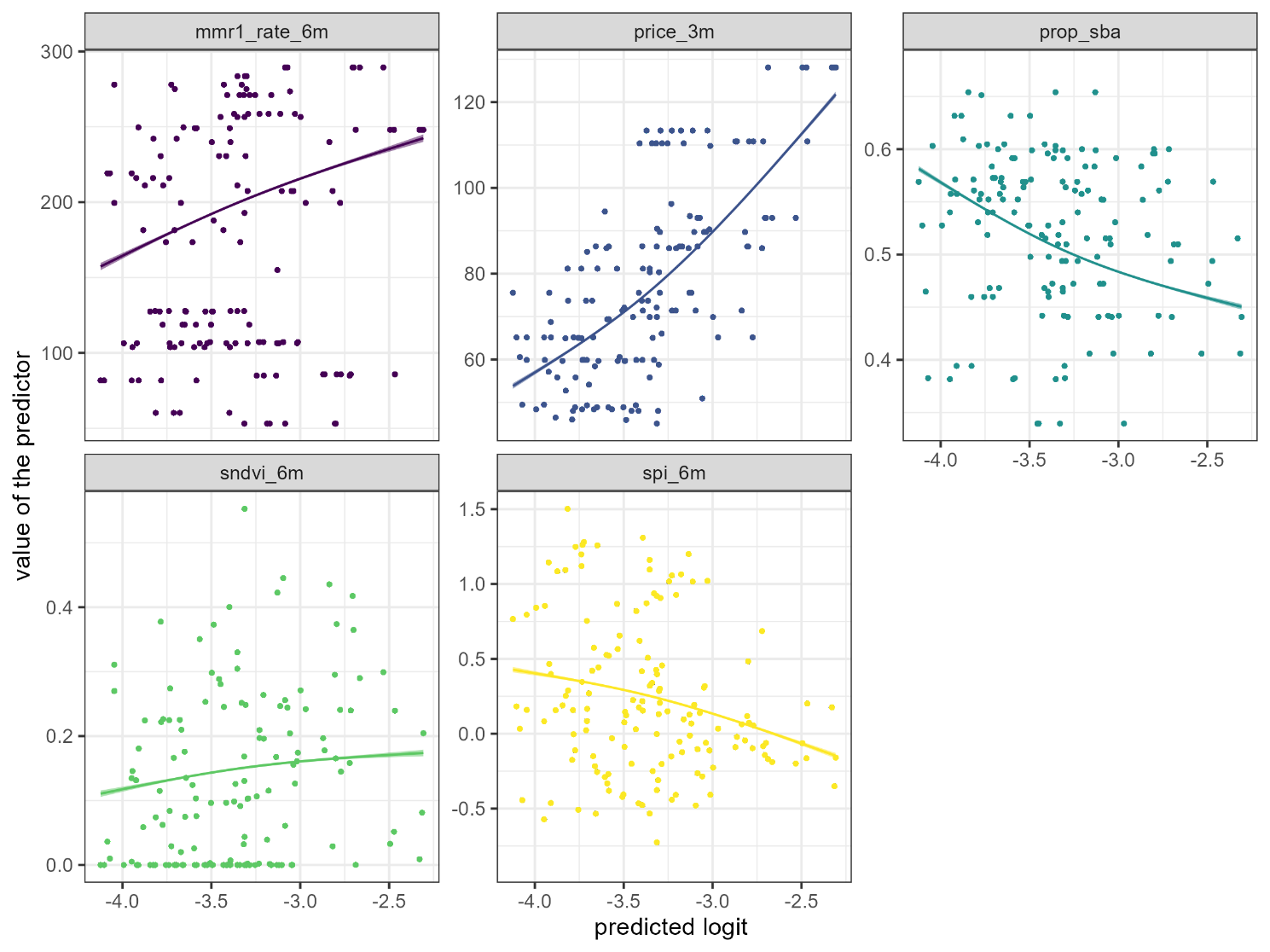


Fig H. Correlation between predicted logits and values of each continuous predictor, for a GLM model of SAM. Lines indicate smoothed trends based on additive models with 3 knots.


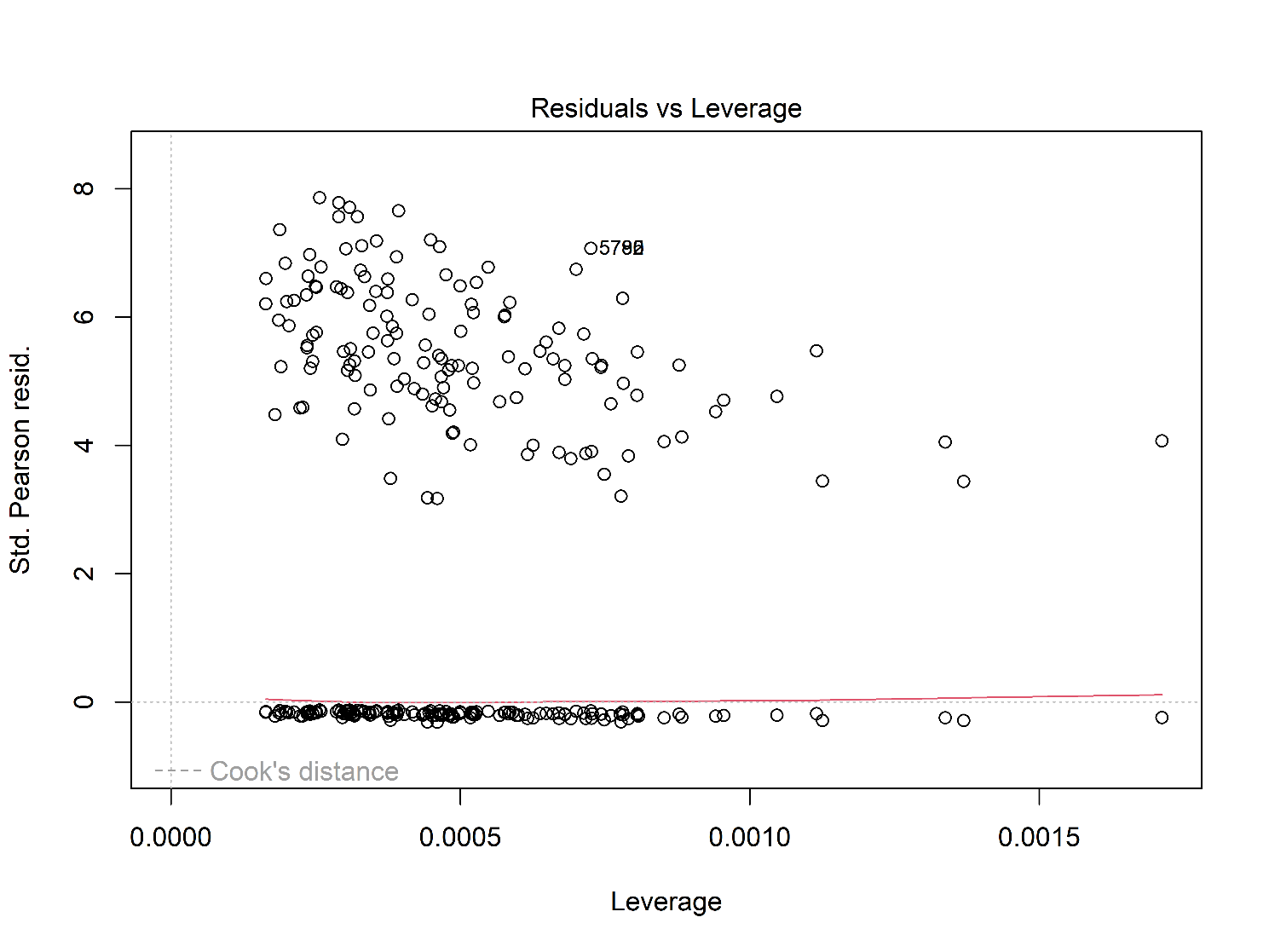


Fig I. Graph of observation leverage versus residuals, for a GLM model of SAM.

Table E. Multicollinearity tests for a GLM model of SAM. Standardised GVIF values ≥ 5 typically suggest collinearity.

| Predictor | Generalised variance inflation factor (GVIF) | Degrees of freedom (Df) | Standardised GVIF GVIF^(1/(2*Df)) |
| --- | --- | --- | --- |
| sndvi_6m | 1.377181 | 1 | 1.173534 |
| mam_admissions_rate_3m_cat | 2.181968 | 3 | 1.138872 |
| price_3m | 3.544109 | 1 | 1.88258 |
| cholera_rate_3m_cat | 9.337423 | 4 | 1.322143 |
| events_rate_3m_cat | 2.041986 | 4 | 1.093343 |
| mmr1_rate_6m | 2.31129 | 1 | 1.520293 |
| prop_sba | 1.930082 | 1 | 1.389274 |
| spi_6m | 1.634515 | 1 | 1.278481 |


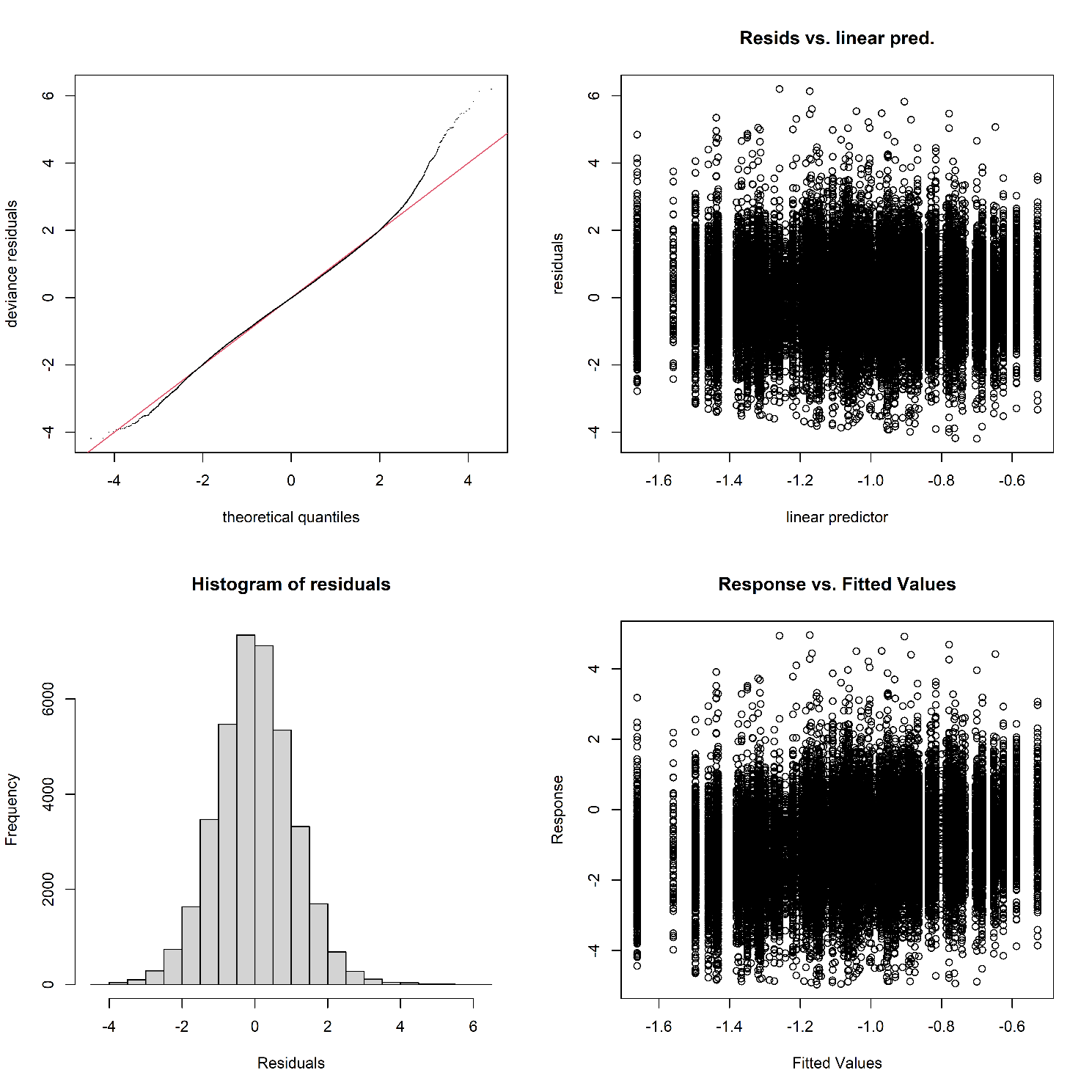


Fig J. Diagnostic plots, for a generalised additive model of WHZ.


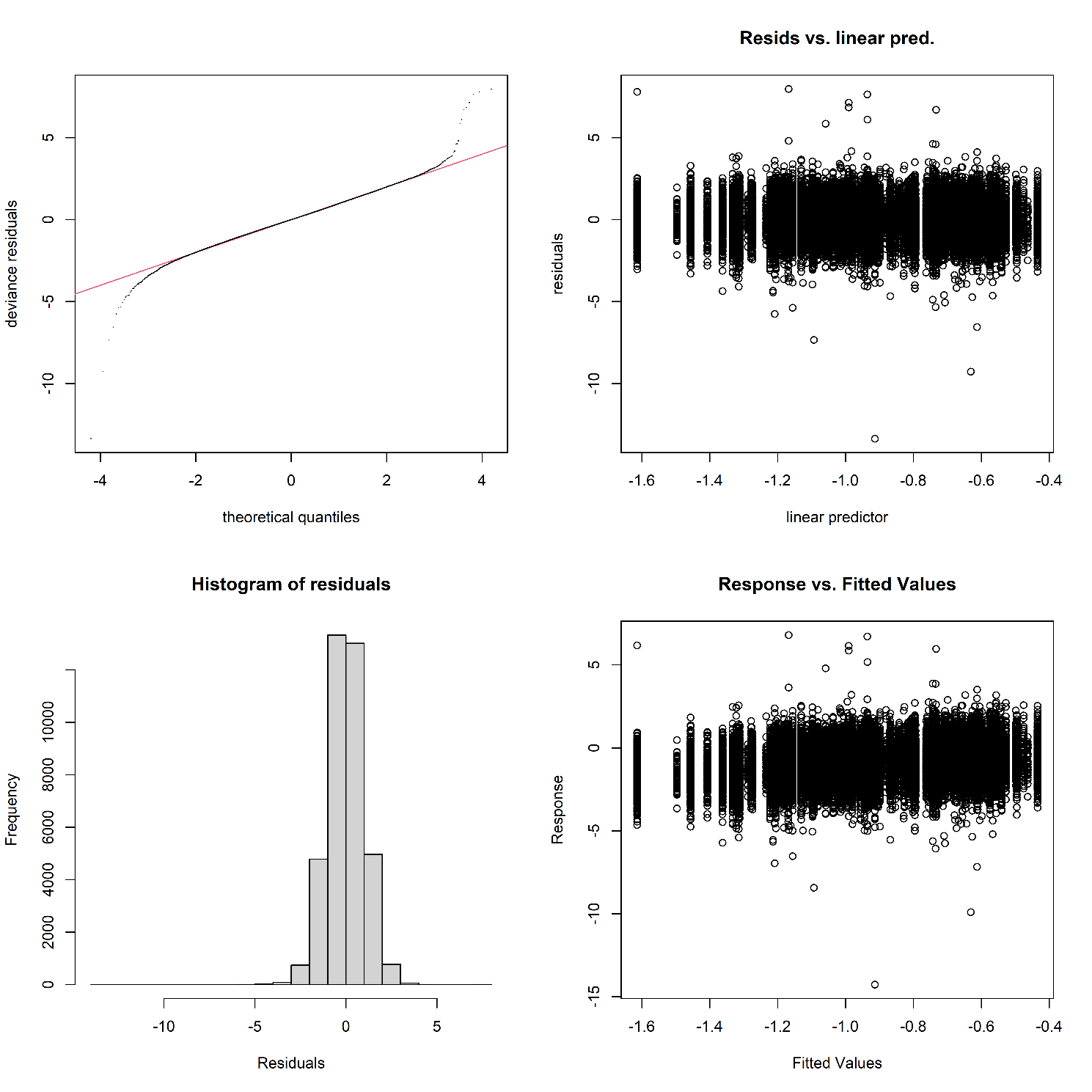


Fig K. Diagnostic plots, for a generalised additive model of MUACZ.


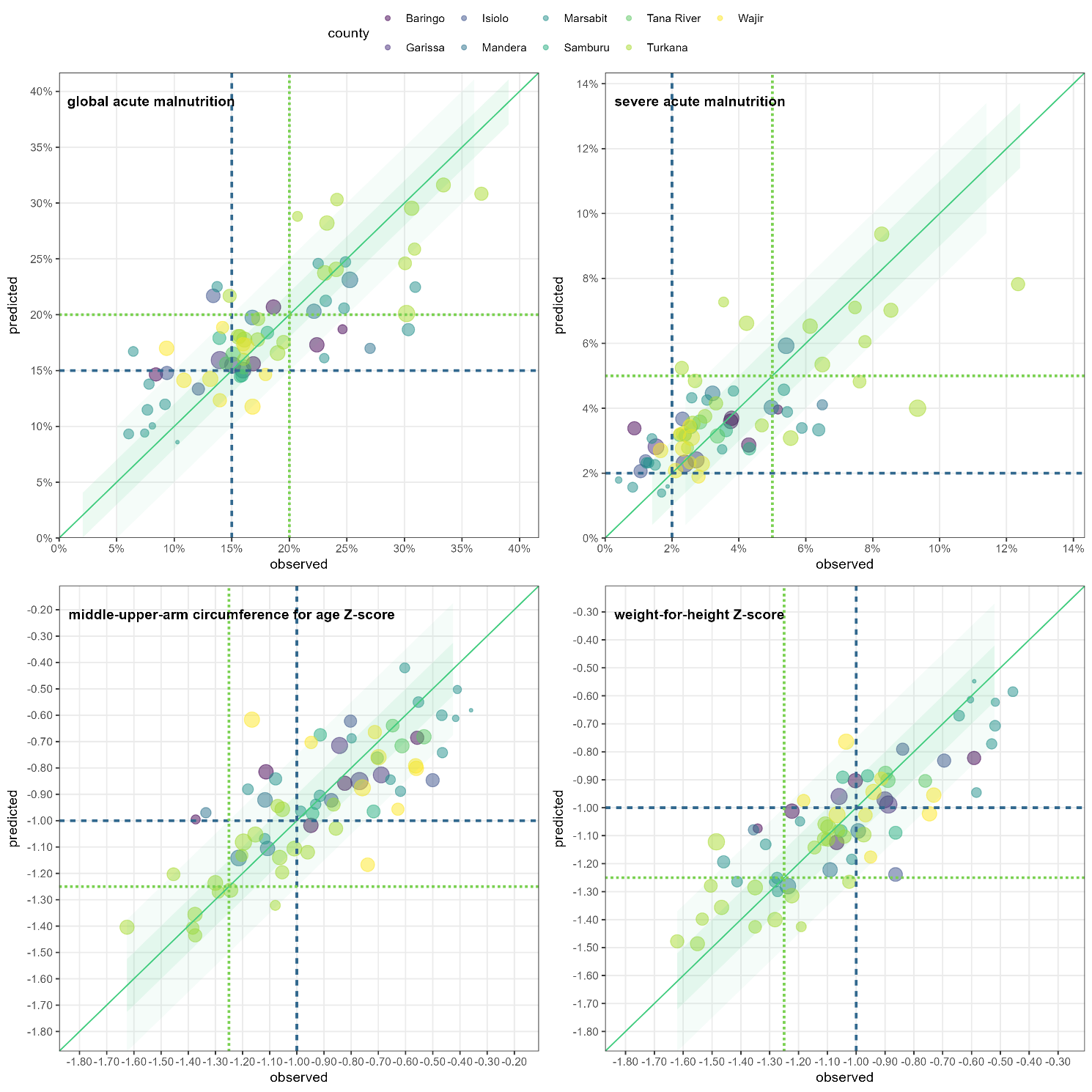


Fig L. Random forest predictions versus observations for each fold in leave-one-out cross-validation, by outcome. Forests were grown by splitting data by up to **five** variables at each tree node. Within each graph, dots represent individual survey strata left out of the training sample and which the model was validated on. The size of each dot is proportional to the number of child-observations, and the colour maps to the county. The diagonal line denotes perfect fit, while shaded areas above and beyond it show alternative error thresholds. Finally, horizontal and vertical lines denote interesting threshold values of the outcome for which the model’s sensitivity was computed.
