# Supplementary material for: Predicting the burden of acute malnutrition in drought-prone regions of Kenya: A statistical analysis
Source: PLOS Glob Public Health. 2026 Apr 10;6(4):e0004485. doi: 10.1371/journal.pgph.0004485 (PMC13068232; doi:10.1371/journal.pgph.0004485)
Supplement: Attachment — Submitted filename: ken_predict_malnut_response_to_reviewers.docx [file pgph.0004485.s003.docx]

| # | Comment | Response | Section/Subsection Changes |
| --- | --- | --- | --- |
| **Editor** | | | |
| E1 | Please insert an Ethics Statement at the beginning of your Methods section, under a subheading ‘Ethics Statement’. | The subheading ‘Ethics Statement’ was moved to the beginning of the Methods section. It states that the study received ethical approval from Amref Ethics and Scientific Review Committee (Ref: ESRC P723/2019) and the LSHTM Ethics Committee (Ref: 15334, Amendment 3). | Methods - Ethics Statement |
| E2 | Amend your detailed Financial Disclosure statement to:   (i) State the initials, alongside each funding source, of each author to receive each grant. (ii) State what role the funders took in the study. If no role: include the sentence “The funders had no role in study design, data collection and analysis, decision to publish, or preparation of the manuscript.” | The Financial Disclosure Statement has been rewritten in full sentences to indicate the initials, alongside each funding source, of each author to receive each grant and clearly state that the funders had no role in study design, data collection and analysis, decision to publish, or preparation of the manuscript | End of manuscript - Financial Disclosure |
| E3 | Provide permissions or evidence of compatible licensing for any reused materials (e.g., maps).  Figure 1:  (a) Provide a direct link to the base layer of the map (e.g. country/region border shape) and include it in the figure legend.  (b) Provide a link to the terms of use/license of the shapefile used.  If shapefile used is from open-access sources like R/ArcGIS, cite the source. | We thank the editor for this reminder. Figure 1 was generated in R using administrative boundary shapefiles obtained from the [Humanitarian Data Exchange (HDX)](https://data.humdata.org/dataset/ken-administrative-boundaries), originally provided by UNOCHA. The shapefiles are distributed under the Creative Commons Attribution 4.0 International (CC BY 4.0) license. We have now included both the direct source link and license information in the figure legend, in compliance with PLOS’s copyright policy. | Figure 1 |
| **Reviewer 1** | | | |
| R1.1 | Line 62-63 - the authors quote a cost for SMART survey as being $10-40,000. However, they themselves have done the analysis of sub-county level SMART surveys. It may be better to compute the actual costs of the SMART surveys that they have actually used instead of stating global national SMART survey costs. | We appreciate this suggestion. The quoted $10–40,000 range was intended as a general reference for SMART survey implementation costs, based on published literature and programme guidance. We agree that using actual estimates from the surveys analysed in this study would provide a more precise and context-specific figure. Unfortunately, detailed costing information for the individual sub-county SMART surveys used was not available to the authors at the time of analysis, as these were implemented by various actors and cost data were not systematically collected or shared.  We have amended the sentence to clarify that the cited cost range is based on global estimates and noted the unavailability of disaggregated cost data for the specific surveys analysed in this study. The revised sentence now reads: “However, they remain somewhat costly to implement, with global estimates ranging from 10-40,000 USD per survey depending on transport, staffing, and other factors” | Background |
| R1.2 | In Line 112, authors refer to causal frameworks in previous studies (References 18 and 22); While the quoted reference 18 and the figures do not refer to the causal frameworks, the reference no. 12 does have a causal framework in page 11 (Figure 2). | We appreciate the reviewer’s close attention to the sources cited. However, we respectfully clarify that Reference 12 (Frison et al., 2016) is a systematic review of anthropometric indicators and does not present a causal framework for acute malnutrition.  In contrast, Reference 18 (Checchi et al., 2022) does include a causal framework, presented in Supplementary Figure S8 (Additional File 1). This framework was developed to inform predictor selection for modelling acute malnutrition in similar contexts and is therefore relevant to the current study.  We have therefore retained Reference 18 in the manuscript as originally cited and believe it is appropriate to support the conceptual basis of our approach. No changes were made to the manuscript text. | None |
| R1.3 | Critical proximate determinants of acute malnutrition include - inadequate dieteray intake and disease (immediate casues), household food insecurity, inadequate care, unhealthy household environment and lack of health services as critical proximate causes. Several of the items in the Table 1 as predictors do not address these critical set of indicators. For example, food security measured through price of food staples is not same as household food insecurity. The study did not include any indicator either direct or proxy on either care or unhealthy household environment. If they had made an attempt, it was possible to get information on some of these from hospitals and health centres in the sub-county level, as well as other surveys possibly at the county level (including e.g. the county level data sheets that are available for DHS-Kenya). The list of predictors in Table 1 are predominantly distal or remote macro-level indicators at least from the perspective of the conceptual framework for acute malnutrition. The authors note that their analysis is in some ways a repetition of what they found elsewhere as published by most of the same authors in South Sudan and Somalia. Compared to those countries, Kenya counties have lot more data from both DHIS2 and other sources on several of the direct or proxy indicators for immediate and underlying causes instead of depending on predominantly basic or remote/macro level indicators as they possibly had to in data poor countries such as Somalia and South Sudan. Authors may wish to clarify why they did not seek to include direct or relevant indicators that can explain the causality better? | We thank the reviewer for this detailed and constructive comment, which raises a crucial point regarding the role of proximate determinants in models of acute malnutrition.  We agree that immediate and underlying causes such as inadequate dietary intake, illness, household food insecurity, poor care practices, and unsafe environments are central to frameworks such as the UNICEF conceptual model and Young (2024) and would indeed be essential for causal inference. However, our modelling objective was not to estimate causal pathways but to optimise predictive performance using data sources that are spatially and temporally aligned with SMART surveys and operationally accessible to humanitarian and government actors. This objective required a careful trade-off between theoretical relevance and data availability across time and geography. Specifically, all predictor variables were required to meet the following criteria:   - Sub-county (or, where unavailable, county) level resolution, consistent with SMART survey strata - Temporal continuity across the study period (2015–2019) - Public availability or sustained partner access, to ensure reproducibility and enable routine application.   Unfortunately, most direct measures of proximate determinants, such as household food insecurity scales, infant and young child feeding practices, or WASH indicators, are derived from household surveys like DHS or MICS. These are typically conducted every 5 years or more, are representative only at national or provincial levels, and were not available for the majority of the SMART survey time points or sub-county clusters used in this analysis. For example, the most recent available DHS for Kenya during our study period was in 2014, and its spatial resolution (county level) and temporal distance from most of our SMART surveys limited its applicability.  We did explore inclusion of administrative data from DHIS2 and county-level health records, in consultation with the Ministry of Health. However, these sources presented substantial limitations:   - Incomplete sub-county disaggregation, particularly for indicators such as child morbidity or WASH access - Inconsistent reporting over time, with frequent missing months or irregular updating - Restricted public access, limiting transparency and reproducibility   We ultimately excluded such indicators to avoid compromising model generalisability and to ensure that all predictors could be realistically applied in a prospective operational setting. Where suitable and consistently available administrative indicators existed at county level (e.g. vaccine uptake), we did include them, as shown in Table 1.  We agree with the reviewer that the reliance on distal indicators such as food prices, vegetation, or literacy constrains the model’s explanatory power and limits interpretation of underlying causal mechanisms. We have now added text in the manuscript to clarify this trade-off. | 1. Methods - Predictors of Malnutrition 2. Discussion- Limitation |
| R1.4 | One should have looked at data related to caring practices, household water and sanitation access, handwashing from closer to the community sources and previous county level reports as well as HMIS data. How would data on for example - proportion of population with access to safe assisted births from "WorldPop" be more valuable than data and information that is available from the counties own records? | We thank the reviewer for raising this point regarding the inclusion of the WorldPop-derived estimate of access to safe assisted births, and the broader concern about our use of global versus administrative data sources.  We included the proportion of the population with access to safe assisted births as a proxy for healthcare access and system functionality, given the consistent association between poor maternal and child health service coverage and undernutrition in children. We acknowledge that this variable, as modelled by WorldPop, is an indirect estimate based on re-analysis of household surveys and not a direct observation. However, it was selected primarily because it met the key inclusion criteria of this study:   - Sub-county level granularity - National coverage - Static or time-aligned with our analysis period - Public availability, enabling reproducibility   While administrative data from county health records or DHIS2 may offer more direct measures of maternal care utilisation, we found such datasets to be incomplete, inconsistently reported, or not publicly accessible at sub-county resolution. In particular, indicators such as facility-based delivery or skilled birth attendance were often missing for several counties or lacked the temporal alignment needed to link with SMART survey rounds. These limitations reduced their feasibility for inclusion in predictive models that aim to be generalisable and operationally replicable.  We fully agree that such administrative sources, if complete and consistently available, could offer valuable alternatives and encourage their further development. We have clarified the rationale for inclusion of the WorldPop variable in the manuscript and acknowledged its limitations in the discussion of predictors and in the limitations section.  Manuscript Changes:  We have updated the Table 1 legend to clarify the rationale behind using WorldPop-derived estimates for safe assisted births. Added in the notes coloumn to this predictor “*Included due to full sub-county coverage and availability during the study period. Used as a proxy for access to maternal health services in the absence of reliable, complete administrative data*”.  In addition, we added the following to the Methods- predictors of malnutrion subsection: “*Where sub-county indicators from administrative sources (e.g. DHIS2) were considered but found to be unavailable, inconsistently reported, or inaccessible, such as skilled birth attendance or facility delivery, we included publicly available modelled estimates (e.g. WorldPop) that provided full spatial coverage and alignment with the study period. These variables were not intended as precise causal measures, but as pragmatic* *proxies for underlying constructs such as access to maternal health services. While we recognise their limitations, they allowed us to maintain comparability across strata and enable operational use in prospective applications.”*  We have also noted this limitation in the Discussion under the relevant paragraph on predictor constraints “*In some cases, where key indicators were conceptually relevant but not consistently available from administrative sources (e.g. DHIS2), we relied on publicly available modelled proxies to preserve coverage and alignment with SMART survey timing. For example, WorldPop-derived estimates of access to safe assisted births were used as a proxy for maternal health service availability, given the lack of reliable sub-county data from health information systems. While these proxies allowed broader spatial modelling and reproducibility, we acknowledge that they may only indirectly capture the intended constructs and could introduce additional measurement error. Their inclusion was a pragmatic compromise to enable scalable, operationally useful prediction, but they should be interpreted with caution.”* | 1. Methods- Predictors of malnutrition- Table 1 2. Methods- Predictors of malnutrition 3. Discussion- limitation |
| R1.5 | A review of the table 1 suggests that data from global sources on indicators which at best can only marginally influence malnutrition outcomes have been given importance somewhat more than other possible indicators which have not even been considered. The authors may wish to clarify the reason for such exclusion of critical indicators and for inclusion of indicators which may have some marginal influence only. | We thank the reviewer for highlighting this concern regarding the reliance on globally modelled indicators that may be distal to the immediate causes of acute malnutrition. We fully agree that the strength of association between such variables and malnutrition outcomes is likely to be weaker than that of more proximal determinants.  As noted in our responses to earlier comments (particularly Comments 3 and 4), our selection of predictors was driven by the practical requirements of sub-county granularity, temporal alignment with SMART surveys, and data availability across the full study period. In several cases, direct indicators of household conditions, care practices, or service access were either unavailable at sufficient resolution or not publicly accessible, leading us to include indirect proxies (e.g. vegetation index, food prices, WorldPop estimates) that could support operational scalability.We have clarified this rationale in the Predictors of Malnutrition subsection of the Methods and in the Limitations section, where we also acknowledge the potential measurement error and limited causal relevance of distal indicators. No further manuscript changes were made in response to this comment, as we believe the concerns were addressed through previous revisions. | None |
| R1.6 | Rest of the paper heavily depends on the clarification that the authors will need to provide, before i can sufficiently comment on the detailed analysis and presenatation as well as on the conclusions of the study based on indicators which do not necessarily fit into the causality framework adequately. | We thank the reviewer for this final comment. We have provided detailed clarifications in response to earlier points, particularly around the rationale for including distal indicators and the exclusion of more direct but unavailable data sources. These clarifications have been incorporated into the Methods and Limitations sections. We hope this addresses the concerns raised and enables a full appraisal of the study’s design, findings, and implications. |  |
