# Supplementary material for: Predicting the burden of acute malnutrition in drought-prone regions of Kenya: A statistical analysis
Source: PLOS Glob Public Health. 2026 Apr 10;6(4):e0004485. doi: 10.1371/journal.pgph.0004485 (PMC13068232; doi:10.1371/journal.pgph.0004485)
Supplement: Attachment — Submitted filename: ken_predict_malnut_response_to_reviewers_2.docx [file pgph.0004485.s004.docx]

PGPH-D-25-00663R1 - Predicting the burden of acute malnutrition in drought-prone regions of Kenya: a statistical analysis

**Revision 2 – Response to reviewer**

| # | Comment | Response | Section/Subsection Changes |
| --- | --- | --- | --- |
| 1 | What was the age range of the children included? | Each survey included the age range 6 to 59 months old. We have now specified this. | Methods, Data sources and Management, Anthropometric Data |
| 2 | The surveys were conducted between January 2015 to December 2019, however, no details have been provided on number of surveys included and the exact dates/month of the year when data were collected. Acute undernutrition is very seasonal, so is drought and rainfall in Kenya/tropical. Having a table showing the list of the surveys, dates and drought periods would be very informative. | We apologise. Possibly through an omission on our part, the paper’s annex (S1 Appendix) was not included in the previous revision that the reviewer looked at. As such, we realise that the reviewer wouldn’t have had access to these details. | S1 Appendix is now uploaded and contains the information requested. Supplementary tables and figures are referred to in the main paper. |
| 3 | While using AIC for initial variable screening is reasonable, relying solely on univariate AIC risks excluding variables that may be weak alone but strong in combination (due to interactions or confounding). Consider discussing this limitation or complementing AIC screening with other selection methods (e.g., lasso, stepwise with interactions, or expert-driven selection). | Yes, agreed. We should note however that we didn’t use AIC to select predictors for consideration, but rather to establish an order in which to enter predictors into the model. Predictors were ultimately excluded only if they did not improve predictive performance on cross-validation.  We have explained the above better and added a note in the Discussion (some text was already there). | Methods, Statistical analysis  Discussion, Limitations |
| 4 | The text mentions "rolling-mean periods" for predictors, but doesn't clarify the time window used (e.g., 1-month, 3-month rolling averages?) or how lags were tested. This is especially critical in modeling nutrition as predictor-outcome relationships are often delayed (e.g., rainfall → harvest → malnutrition). | 3- and 6-month averages, as mentioned in the Methods.  We then selected the best-fitting of the alternative average periods based on AIC, as shown in the S1 Appendix, now better signposted in the main text. | Methods, Data sources and management, Predictors of malnutrition  Methods, Statistical analysis  S1 Appendix |
| 5 | The stepwise addition of variables based on AIC is common but susceptible to overfitting, especially without regularization. Was any constraint placed on the maximum number of predictors to avoid overly complex models? | No, but as noted in the Methods, Statistical analysis, each candidate model was evaluated based on its cross-validation performance, which should capture overfitting. Stepwise addition was monitored through cross-validation. | No change. |
| 6 | For the random forests: using only 3 variables per split node is quite low — why was this chosen? Did you test performance with different values? | We tested performance with 5 variables, but did not see any discernible improvement in performance (certainly not to the extent that the resulting predictions would be operationally useful). We have now signposted these additional results in the main text and included them in S1 Appendix. | Results, Predictive model performance, Random forest models  S1 Appendix |
| 7 | Were assumptions of the GLMs/GAMs assessed (e.g., residual distribution, homoscedasticity for continuous outcomes)? Some brief mention would strengthen confidence in model validity. | Yes, they were. We have now explained which diagnostics were done, and results are included in S1 Appendix. | Methods, Statistical analysis  S1 Appendix |
| 8 | It’s unclear how “alternative crisis severity thresholds” were defined (e.g., standard IPC thresholds for GAM >10%, 15%?) lines 171/172. Including these definitions and rationales would aid interpretability. | As the reviewer notes, these are standard thresholds used by the IPC. We have clarified this and added citations. | Methods, Statistical analysis |
| 9 | Were the random forest models formally compared with GLMs model? Or how was `marginally better’ line 254 comments based on? | No, just visually. We have clarified this in the text. | Results, Predictive model performance, Random forest models |
| 10 | The authors notes poor performance of the models. Any thoughts on why the model poorly performed and alternatives would greatly improve the model. | We think we have already made some suggestions in the Discussion, Limitations section. | No change. |
