# Supplementary material for: Predicting the burden of acute malnutrition in drought-prone regions of Kenya: A statistical analysis
Source: PLOS Glob Public Health. 2026 Apr 10;6(4):e0004485. doi: 10.1371/journal.pgph.0004485 (PMC13068232; doi:10.1371/journal.pgph.0004485)
Supplement: Attachment — Submitted filename: ken_predict_malnut_response_to_reviewers_3.docx [file pgph.0004485.s005.docx]

PGPH-D-25-00663R2 - Predicting the burden of acute malnutrition in drought-prone regions of Kenya: a statistical analysis

**Revision 3 – Response to reviewers**

**Reviewer #3:**

This is a strong and well-thought study that makes a valuable contribution to the literature on nutrition surveillance in drought-affected countries. The cautious interpretation of findings and emphasis on operational relevance are appropriate. Minor revisions could further strengthen the manuscript, including brief clarification of how the models might be used in practice for early warning or situational awareness and light editing for conciseness. Overall, the manuscript is suitable for publication after minor revisions.

ANS. Thank you. We have further edited the Discussion (Conclusions) to clarify whether we think the models can be used in practice (in short, no). We’ll defer to the editor as regards any needed word count reduction.

**Reviewer #4:**

This study evaluates predictive approaches, namely generalized linear and additive models as well as random forest methods, to estimate acute malnutrition using routinely collected and/or publicly available data in combination with SMART survey data in Kenya. The analysis covers 2015–2019, a period that included one major drought episode. The authors examine global acute malnutrition (GAM), severe acute malnutrition (SAM), middle-upper-arm circumference-for-age Z-score, and weight-for-height Z-score as outcomes. Predictors were selected using AIC criteria, and model performance was evaluated using cross-validation. Overall, predictive performance was moderate, with the most promising results obtained from the random forest model for GAM.

The rationale of the study is highly compelling. The possibility of using routinely collected or publicly available data in drought-prone settings to estimate malnutrition burden, rather than relying on educated guesses or costly field surveys, is of clear operational importance. The manuscript is part of a series of studies attempting to predict malnutrition burden, and in this Kenyan setting the authors had access to more extensive and granular data than in previous applications. Despite this, predictive accuracy remains modest, which in itself is an important and informative finding.

In my view, the study is highly relevant. The authors have addressed previous reviewer comments carefully and have added clarifications that substantially improve transparency, particularly regarding predictor selection, proxy measures, and trade-offs between conceptual relevance and operational feasibility. The discussion of limitations is candid and balanced. The manuscript could still be made slightly more accessible for readers less familiar with predictive modelling, for example by briefly clarifying the rationale for AIC-based model selection and the interpretation of cross-validation results. However, the current version already does a solid job in guiding the reader through the analytical strategy.

ANS. We have added clarifications on AIC-based selection and a brief explanation of cross-validation to the Methods, Statistical Analysis.

The strongest limitation, as acknowledged by the authors, is that the study period includes only one major drought. This necessarily restricts variability in the outcome and limits the ability to assess whether predictor–outcome relationships would remain stable across multiple drought cycles. In this light, it would be helpful if the authors could clarify why more recent data, particularly from the 2022–2023 drought, were not incorporated. Even if data availability or compatibility constraints prevented this, a short explanation would strengthen the manuscript.

ANS. We have now mentioned this point within the Discussion, Limitations. Briefly, the study was commissioned by Unicef and approved by the Ministry of Health only for the analysis period presented in this paper. The analysis and write-up was heavily delayed due to the pandemic and a number of personal circumstances.

With respect to modelling strategy, the authors rely on AIC-based selection for generalized models and compare these with random forest approaches. This is a reasonable and transparent strategy, particularly given the modest sample size and the operational nature of the research question. One additional sentence elaborating on why penalized regression approaches such as lasso were not pursued could be helpful, especially since such methods are often considered when handling multiple correlated predictors. That said, the authors’ argument that alternative machine learning methods would likely face similar structural constraints, given the limited temporal variability and measurement error in predictors, is plausible and does not detract from the core findings.

ANS. Thank you. We have added a sentence to the Discussion, Limitations.

The cross-validation approach at the survey-stratum level is appropriate and clearly described. Readers may benefit from a brief reflection in the discussion on how predictive performance might differ under alternative validation schemes, for example temporal holdout designs, although this is not essential for publication.

Overall, this study makes a meaningful contribution to the literature on predictive modelling of malnutrition burden. Importantly, it demonstrates both the potential and the current limits of such approaches when constrained to routinely available data in real-world humanitarian settings. The manuscript does not overstate its findings and appropriately frames them as part of an ongoing effort to improve predictive tools. I believe the study is suitable for publication and will be of interest to researchers and practitioners working at the intersection of nutrition surveillance, humanitarian response, and predictive analytics.
